# Is It Time to Say Farewell to the ISDS System? Comment on "The Trans-Pacific Partnership: Is It Everything We Feared for Health?"

**DOI:** 10.15171/ijhpm.2016.125

**Published:** 2016-09-13

**Authors:** Raphael Lencucha

**Affiliations:** School of Physical and Occupational Therapy, McGill University, Montreal, QC, Canada.

**Keywords:** Trans-Pacific Partnership (TPP) Agreement, Investor-State Dispute Settlement (ISDS), Health Policy, Government Regulation

## Abstract

Investor-state dispute settlement (ISDS) continues to plague health-oriented government regulation. This is particularly reflected in recent challenges to tobacco control measures through bilateral investment agreements. There are numerous reform proposals circulating within the public health community. However, I suggest that perhaps it is time for the community to explore a stronger position on ISDS. I draw from mounting evidence on the problematic uses of the ISDS to explore the proposition that ISDS is no longer justified. I tackle the normative question of should the ISDS system persist and point out that the ISDS system is not justifiable from a development perspective and because of its nefarious use, is of no added value to a system that could rely on domestic courts.

## Introduction

Investor-state dispute settlement (ISDS) provisions have existed since the 1960s. However, it was not until 1987 that the first dispute was filed. Since then there has been an explosion in the number of cases. According to the United Nations Conference on Trade and Development (UNCTAD) 2016 World Investment Report, a record 70 disputes were initiated in 2015, bringing the total number of known disputes to 696.^1^ The ISDS provision is designed to provide an added level of security to protect investors from expropriation (direct and indirect), nationalization and other forms of discrimination. It is argued that this protection is needed in countries with weak rule of law and unpredictable political orders; a transnational safety valve. From this perspective the ISDS provision is viewed as a valuable tool to attract foreign investment and protect investors in “risky” environments. Despite the apparent virtues of ISDS it has become one of the most controversial aspects of the international investment regime. The problems with ISDS are conditioned by the rapid rise in the number of international investment agreements (IIAs) in the past two decades. As of 2015, there were 3304 IIAs in existence.^1^ Each agreement provides a unique forum for investors to file a dispute. The rapid and sustained rise in the number of challenges and the nature of these challenges is what is so troubling. It is not surprising that Labonté and colleagues have identified ISDS as one of the pressing potential risks embedded in the Trans-Pacific Partnership (TPP).^2^ Unlike the authors, I suggest that perhaps it is time for the health community to explore a stronger position on ISDS. I draw from mounting evidence on the problematic uses of the ISDS to explore the proposition that ISDS is no longer justified. In arguing this controversial point, I recognize that there are numerous major obstacles to realizing this position. Most commentators have chosen a more path dependent approach. They recognize that the system is broken, even fundamentally flawed, but acquiesce to the fact that ISDS is so embedded in the economic landscape that one must accept that, at least in the medium-term, the system will persist. I tackle the normative question of *should* the ISDS system persist and point out that the ISDS system is not justifiable from a development perspective and because of its nefarious use, is of no added value to a system that could rely on domestic courts.

## Investor-State Dispute Settlement and Development

To begin, there is mixed evidence to support the hypothesis that investment agreements do indeed lead to greater foreign investment, which is arguably the central purpose of such agreements.^3,4^ Not surprisingly, evidence suggests that the domestic institutional environment (eg, low levels of corruption, strong property rights protection) is a key factor in attracting foreign direct investment (FDI).^5^ Rose-Ackerman and Tobin found that investment agreements only had a positive impact on facilitating investment in stable rule-of-law countries, which contradicts the logic that ISDS will facilitate investment in low rule-of-law countries.^6^ Even where risk of expropriation reduces investment there is evidence to suggest that other factors such as foreign aid can mitigate the adverse effect of this risk.^7^ Market openness and investment incentives are also found to play a key role in attracting investment independent of international agreements. Not surprisingly the potential for profit, through such aspects as commodity price, plays a powerful role independent of the institutional environment. Lee provides a dramatic example of how commodity price (ie, oil price) can even be increased due to unstable political contexts (ie, conflict) inducing investment.^8^ Together this evidence supports one commentators point that “if host countries are committed to trying to attract more FDI, bilateral investment treaties (BITs) have not provided a short-cut from the need to implement broader reforms of domestic institutions.”^9^ The fact that FDI does not hinge on investment agreements undercuts the argument that ISDS is necessary for economic development.

## Nefarious Use of Investor-State Dispute Settlement

It is true that the ISDS system does provide a venue for investors to seek compensation for expropriation, nationalization or other discriminatory practices. However, because abuses of the system are almost impossible to control it can be argued that the costs outweigh the benefits. This point is particularly salient in light of the fact that investors will likely keep investing in the absence of ISDS due to factors presented above. To examine cost, it is important to examine the nature of the investor-state disputes. Pelc analyzed claims filed between 1993 and 2015.^10^ He found that:

*“Most disputes today are not over direct takings, but over indirect expropriation. And most respondent-countries are not rent-seeking regimes with low rule-of-law, but stable democracies with independent judiciaries. To put it in stark terms, the greatest portion of legal challenges in the investment regime today seek monetary compensation for regulatory measures implemented by democracies”* (p 2).

In this timeframe, only 17% of ISDS claims were filed because of direct expropriation. This pattern points to the fact that the ISDS system is primarily being used to challenge government regulation. For example, of the 70 cases brought forward in 2015, more than 20 were brought against legislative reforms in the renewable energy sector alone.^1^

This evidence supports the criticisms of ISDS voiced by the public health community which rest on the claim that firms are using ISDS to challenge legitimate regulatory measures.^11,12^ They further argue that these cases are being filed not with the ultimate goal to gain compensation but to discourage other countries from imposing such measures (otherwise referred to as “regulatory chill”).^13^ Pelc finds that investors only win 10% of indirect expropriation claims when brought against democratic countries, which suggests that, if firms are truly rational entities, cases are being brought forward to achieve peripheral objectives. For example, Pelc notes that these cases rarely reach a settlement and are widely publicized meaning that firms want to extend the length of the dispute and publicize the process in order to signal to other countries that a similar protracted legal dispute awaits them if they choose to regulate.^10^ These findings align well with recent cases filed by tobacco firms under the ISDS system. For example, Philip Morris challenged Australia’s standardized tobacco packaging legislation using a BIT signed between Hong Kong and Australia. Philip Morris Asia had restructured its ownership to establish its presence in Hong Kong, in what appeared to be a maneuver to utilize the BIT as a forum to file a dispute against Australia. The tribunal recognized this maneuver as such and dismissed the case on jurisdictional grounds. Another case filed by Philip Morris International (PMI) against Uruguay in 2010 was finally resolved in favor of Uruguay in 2016 and even though PMI lost the case, the monetary cost incurred by Uruguay prior to the decision was substantial. The cost was so significant that international civil society organizations rallied to generate a legal fund, to ensure that Uruguay did not have to settle the case or withdraw the tobacco control measures being challenged.^14^ In the end the tribunal required PMI to pay Uruguay’s legal fees, however, this decision cannot be expected in all cases. The average cost of an ISDS case is US$8 million with an upper range of US$30 million.^15^ This cost does not include the award of compensation which averages US$10 million.^15^ The procedural cost is estimated to be five times more than a state-state dispute within the World Trade Organization (WTO) dispute settlement system.^10^

One of the challenges with a “reform” position is that solutions remain elusive and ultimately frivolous cases are impossible to preempt. One proposed solution is a voluntary carve-out of tobacco from disputes under the TPP. Civil society organizations lobbied vigorously to have tobacco excluded from the ISDS chapter of the TPP in an attempt to quash future tobacco-related investor-state disputes. The exclusion of tobacco-related disputes in the TPP agreement was controversial even within the health community. First, despite the efforts to establish a “carve-out” the eventual text requires that countries opt-in to the exclusion of tobacco, meaning that some countries may choose to opt-out. In addition, the countries that chose to opt-in remain vulnerable to tobacco-related disputes under other investment agreements (the “forum-shopping” problem). Pendas and Mathison point out that TPP members are party to almost forty IIAs and note that the text “suggests that previously signed (agreements) will then coexist, with the TPP Investment Chapter … trigger(ing) the potential for forum shopping for certain investors” (p 161).^16^ Third, if ISDS is problematic for tobacco control, then it is likely problematic for governments attempting to regulate alcohol or unhealthy foods. Thus, some argue that tobacco “exceptionalism” ignores more general risks to regulation. While the tobacco “carve-out” symbolizes a general shift towards the recognition that governments must have the space to regulate in the public interest it also highlights the difficulty addressing the problematic aspects of the ISDS system. In other words, the proliferation of overlapping IIAs limits the value of making exceptions for one product in one agreement without attending to more systemic issues.

It is clear that certain reforms could improve upon the existing ISDS system. Structural limitations of the current system include a lack of transparency (ie, prevents public access to and scrutiny of proceedings) and is costly.^11^ However, the central and irreparable dimension of ISDS is how firms use the system to achieve peripheral ends. Firms are profit maximizing and regulation is a threat to this bottom-line. Large firms have the capacity to use the system as a strategic tool to dissuade regulation, even if they know they will likely not “win” the case. The position I am taking does not discount the fact that investors must be able to seek remedy for legitimate grievances. The benefit of having domestic courts take on the sole responsibility for investment disputes is that it would immediately eliminate the duplication of legal costs incurred by states. For example, all of the tobacco-related investment disputes have involved domestic litigation in addition to claims filed through the ISDS system.

A common rationale used to argue for ISDS reform and more recently its elimination is that states must have the sovereign authority to regulate in the public interest.^17^ This is also a common rationale expressed by the public health community in response to challenges brought forward against tobacco regulation at the WTO. This type of argument fails to address the nuance of the contemporary context of global governance. In fact, investment agreements clearly permit states to regulate in the public interest and moreover the legality of investment agreements is derived from the fact that states consent to the provisions. It is important to recognize that sovereign states are known to behave in discriminatory and unlawful ways and should remain accountable to contractual agreements. If the public health community is to take up the position that ISDS must be eliminated, it will be important to develop more robust arguments that integrate nuance. The thrust of my argument is that domestic courts should serve this function and if domestic legal institutions cannot be trusted to provide this service then investors must be accountable for the risks taken.

I anticipate that some may argue that the very existence of the ISDS system is premised on the fact that many domestic legal institutions cannot be trusted to rule impartially. In light of the information provided above, if stable and reliable domestic courts are relied upon to protect investor rights then states have incentive to construct strong rule of law. In other words, we might anticipate that the elimination of ISDS might create greater incentive to develop strong domestic institutions to attract investment. As noted above, the domestic institutional context has always been important to attract investment. One might also argue that if I am suggesting ISDS be eliminated then why not argue for the elimination of the WTO dispute settlement system, since many of the policy issues that lead to investment disputes also lead to trade disputes. I would respond by pointing out that the WTO dispute settlement understanding is a public international law institution that is oriented towards aligning domestic policy with the rules of the WTO agreements. This system is not designed to compensate firms, creating different incentives for dispute. Although dispute brought through the WTO system still brings procedural costs it is worth returning to the point that on average the procedural costs are five times higher in the ISDS system.

## Conclusion

Labonté and colleagues highlight one improvement embedded in the text of the TPP, which is that “investor’s expectations of future earnings alone are insufficient cause for a claim.”^2^ Pendas and Mathison note that the TPP elevates the standard for indirect expropriation whereby measures taken to “protect legitimate public welfare objectives, such as public health, safety and the environment” are permitted.^16^ Again, the problem with a reform agenda is that no provision will preempt the filing of a frivolous dispute. As noted earlier, the legal merit of the case seems to be secondary to the use of ISDS to achieve peripheral ends. In this vein, Labonté and colleagues note, the TPP still does not go far enough in its structural changes. However, I am suggesting that maybe it is time for the public health community to explore a stronger position. To conclude, it is clear that firms continue to abuse the ISDS system by filing frivolous cases intended to create a hostile regulatory environment. Unfortunately, there are no simple solutions to this problem. The elimination of the ISDS system with a corresponding and systemic effort to create a global economy that is supported by just domestic legal institutions seems to be a reasonable position to explore.

## Ethical issues

Not applicable.

## Competing interests

Author declares that he has no competing interests.

## Author’s contribution

RL is the single author of the paper.
